# Targeting GITR in cancer immunotherapy – there is no perfect knowledge

**DOI:** 10.18632/oncotarget.28461

**Published:** 2023-06-19

**Authors:** Diwakar Davar, Roberta Zappasodi

**Affiliations:** ^1^Hillman Cancer Center, University of Pittsburgh Medical Center (UPMC), Pittsburgh, PA 15232, USA; ^2^University of Pittsburgh, Pittsburgh, PA 15232, USA; ^3^Division of Hematology and Medical Oncology, Department of Medicine, Weill Cornell Medical College, NY 10065, USA; ^4^Immunology and Microbial Pathogenesis Program, Weill Cornell Graduate School of Medical Sciences, NY 10065, USA

**Keywords:** cancer, immunotherapy, programmed death-1 (PD-1), cytotoxic T-lymphocyte Antigen-4 (CTLA-4), glucocorticoid-induced TNFR-related protein (GITR)

## Abstract

Glucocorticoid-induced TNFR-related protein (GITR) belongs to the TNFR superfamily (TNFRSF) and stimulates both the acquired and innate immunity. GITR is broadly expressed on immune cells, particularly regulatory T cells (Tregs) and natural killer (NK) cells. Given its potential to promote T effector function and impede Treg immune suppression, GITR is an attractive target for cancer immunotherapy. Preclinically, GITR agonists have demonstrated potent anti-tumor efficacy singly and in combination with a variety of agents, including PD-1 blockade. Multiple GITR agonists have been advanced into the clinic, although the experience with these agents has been disappointing. Recent mechanistic insights into the roles of antibody structure, valency, and Fc functionality in mediating anti-tumor efficacy may explain some of the apparent inconsistency or discordance between preclinical data and observed clinical efficacy.

## INTRODUCTION

The glucocorticoid-induced tumor-necrosis factor receptor related protein (GITR, also known as TNFRSF18, AITR, and CD357) belongs to the TNF superfamily, which comprises 19 ligands and 29 receptors [1]. TNF superfamily (TNFSF) ligands and TNF receptor superfamily (TNFRSF) receptors are expressed by a wide variety of immune cells including T cells, antigen-presenting cells (APC), and tumor cells [2, 3]. The diverse expression pattern of TNFSF/TNFRSF proteins explains the critical role they play in coordinating immune responses. In humans, GITR is constitutively expressed on Foxp3+ regulatory T cells (Treg) at high levels and at lower levels on CD56+ natural killer cells, B cells, naïve, and memory T cells [4–6]. Upon T cell activation, GITR expression is upregulated on both Tregs and CD4+ and CD8+ effector T (Teff) cells [4–6].

TNFSF ligands are type II membrane proteins comprising a C-terminal TNF homology domain (THD) separated from the cytoplasmic domain by a stalk region of variable length [7, 8]. TNFSF ligands are typically membrane bound, although soluble moieties occur naturally due to proteolytic processing in the stalk region separating the THD from the transmembrane domain [9]. Human TNFRSF receptor activation requires trimerization and membrane-bound ligand trimers trigger receptor signaling more efficiently compared to the soluble, cleaved moieties. TNFRSF receptors are type I transmembrane proteins that contain between 1 and 6 pseudorepeats of cysteine-rich domains (CRDs) in their ectodomains, which are involved in ligand binding but can also promote receptor self-assembly [10, 11]. The natural GITR ligand (GITRL, TNFSF18) is predominantly expressed by activated APCs, including dendritic cells, macrophages, and activated B cells [12, 13]. GITRL expression can also be induced in endothelial cells following type I interferon (IFN) exposure [4] and has been observed in certain tumor types (e.g. gastrointestinal cancers and myeloid cell neoplasms) [14, 15].

TNFRSF members possess a domain that mediates self-assembly of two (or three) receptor molecules creating a single high affinity site for non-covalent binding of TNFSF ligand trimers [16, 17]. From a signaling perspective, the majority of TNFRSF members – including GITR – contain motifs that bind to TNF receptor-associated factor (TRAF) that links these receptors to intracellular signaling pathways including nuclear factor-κB (NF-κB), JUN N-terminal kinase, and mitogen-activated protein kinase (MAPK) pathways [18, 19]. Like other TNFRSF members, GITR lacks intrinsic enzymatic activity, and upon ligation through GITRL, GITR signaling is mediated by TRAF5 and TRAF2 protein adaptors to induce the NF-κB and MAPK pathways, which leads to the upregulation of critical cytokines for T cell activation and proliferation (IL-2 and IFN-γ) [20, 21]. In contrast, the GITRL reverse signaling in APCs and tumor cells was reported to promote some tolerogenic effects, including the release of TGF-beta, IL-10, and induction of indoleamine 2,3-dioxygenase (IDO) for tryptophan catabolism [14, 15, 22].

Because of the overall and multiple positive effects on T cell responses, GITR has emerged as a promising immunotherapeutic target, similar to other TNFRSF members, including in particular 4-1BB (CD137/TNFRSF9), and OX40 (CD134/TNFRSF4) [23, 24]. Several preclinical studies have demonstrated strong anti-tumor activity of GITR stimulation using either agonist antibodies (Abs) or multimeric GITRLs as monotherapy or in combination with other types of immunotherapy, including vaccines and immune checkpoint inhibitors (ICI), against multiple syngeneic tumor models. This led to the clinical development of human GITR agonist agents. Since the first anti-human GITR agonist Ab (TRX518) entered the clinical evaluation in 2010, 11 additional human GITR agonist agents have been tested in the clinic. Despite the huge expectation based on the extremely supportive preclinical data, the efficacy of human GITR agonists has been limited in patients. This may reflect the clinical scenario in which historically these agents have entered the clinical evaluation for therapeutic efficacy – mainly in patients with immunotherapy-refractory advanced tumors. Alternatively, this outcome may be due to fundamental biologic differences of the GITR pathway in the human and mouse systems. Here, we discuss these aspects, with a focus on the clinical landscape of GITR agonists and how the field has evolved to guide the development of clever modalities to engage the GITR pathway for the treatment of cancer in patients.

### Rationale for targeting GITR in cancer

The rationale for targeting GITR can be separately considered through its effects upon either the Teff or Treg cell compartments [25, 26]. In the context of Teff cells, GITR activation increases Teff cell cytotoxic function [27], and activation by inducing IL-2 and IFN-γ, enhancing CD25 expression, in turn stimulating cell proliferation [28, 29]. In addition, GITR ligation promotes T cell survival for example of bone marrow-derived CD8+ memory T cells, at least partially by upregulating expression of Bcl-xL and separately by reducing T cell apoptosis [21, 30]. The effects of GITR signaling upon Tregs are more complex. *In vitro* studies demonstrated that GITR stimulation can reduce Treg immunosuppression via two main mechanisms, (1) by protecting Teff from Treg-mediated inhibition [31], and (2) by directly reducing Treg suppression activity [32, 33]. *In vivo*, agonistic anti-GITR monoclonal Abs (mAbs), such as the anti-mouse GITR DTA-1 and the anti-human GITR MK-4166, were found to transiently increase intratumoral Treg proliferation and activation, although these cells eventually became unstable and were preferentially targeted for elimination [34, 35]. In fact, GITR can be exploited as a target to deplete Tregs using Abs that are able to bind with high affinity to Fc receptors, as DTA-1 and MK-4166. Intratumoral Tregs express GITR at relatively high level, making GITR a suitable marker for preferential intratumoral Treg targeting, which is highly desired to avoid systemic autoimmune toxicity. Preclinical *in vivo* studies have shown that the anti-tumor activity of GITR agonist Abs is largely dependent on this mechanism of Treg depletion [36], although functional Treg modulation can also contribute to the anti-tumor immune responses [37]. The extensive perturbation of the Treg compartment in the tumor microenvironment with GITR agonist mAbs has shown to in turn promote functional reinvigoration of CD8+ T cells [35]. In addition, it was reported that direct GITR stimulation in CD8+ T cells induces extensive metabolic changes supporting CD8 T cell proliferation and effector function [38]. These multiple positive effects in T cell responses may explain the potent T cell costimulatory activity and antitumor efficacy observed with the anti-GITR DTA-1 Ab in several mouse syngeneic tumor models, including CT26 and MC38 colorectal cancer [35, 39], B16 melanoma [40], C3 cervical cancer [41], and multiple models of glioblastoma [34]. In preclinical models, the antitumor efficacy of DTA-1 was found to be synergistic with anti-CTLA-4 and PD-1 [32, 38, 42–44], but not with anti-CD25, likely secondary to anti-CD25-mediated depletion of Tregs and Teff cells, both of which express CD25 [39].

Taken collectively, these preclinical findings in mouse syngeneic models indicate the GITR targeting results in enhanced Teff function, and induces potent anti-tumor efficacy – dependent upon both agonistic GITR signaling and Treg modulation – as neither mechanism singly fully rescues anti-tumor T cell responses [34, 35, 40]. Overall, the above observations provided compelling rationale to evaluate GITR agonists in cancer patients.

### Clinical development of GITR targeting mAb: form meets function

GITR therapeutic targeting requires careful consideration of the complex structural and mechanistic features of GITR:GITRL interactions. Given the observed preclinical efficacy of DTA-1, the initial clinical development of GITR agonists focused on monospecific agonistic mAbs, although more recently bispecific agonistic mAbs, and co-stimulatory GITR ligands have been developed.

Nine GITR monospecific agonistic mAbs – AMG-228, ASP1951, BMS-986156, GWN323, INCAGN1876, MK-1248, MK-4166, REGN6569, and TRX518 – have been publicly disclosed, and the structure, isotype, predicted antibody-dependent cellular cytotoxicity (ADCC) and valency of these mAbs are summarized in Table 1. Generally, regardless of structure, the 7 GITR agonists (AMG-228, BMS-986156, GWN323, MEDI1873, MK-4166, MK-1248, and TRX518) studied as a monotherapy or in combination with PD-1 inhibitors or chemotherapeutic agents in patients with advanced solid tumors demonstrated no unusual safety signals [45–51]. Of these, only TRX518 reported single agent activity (1 responder with PD-1 and CTLA-4 refractory hepatocellular carcinoma) [46]. Of note, TRX518 is the only agent that is known to block the interaction between GITR and GITRL thus abrogating the potential GITRL reverse tolerogenic signaling while triggering GITR co-stimulation. Combinations of GITR with anti-PD-1 immunotherapy resulted in clinical responses, although without additive toxicity, and chemotherapy combinations were uninformative. Pharmacodynamic effects observed in these studies included extensive modulation of peripheral Tregs, with reductions in GITR+ Tregs and effector Treg subsets, and trends toward increased proliferating CD8 Teffs and NK cells with combination therapy [32, 52]. Interestingly, Treg reductions were observed also with Fc-inert GITR agonist mAbs (such as TRX518), indicating that functional modulation of Tregs through GITR signaling can take place in humans. Despite the poor efficacy outcome with agonist GITR agents, three of them (ASP1951, INCAGN1876 and REGN6569) remain in clinical development, of which only REGN6569 has been reported on publicly [53]. The clinical efficacy is summarized in Table 2.

**Table 1:** Structure of GITR agonists being evaluated in ongoing or completed clinical trials

Agent (Sponsor)	Sponsor	Structure	Isotype	Predicted ADCC	Valency
AMG-228	Amgen	Monospecific agonistic antibody	Humanized IgG4	No	Tetravalent
ASP1951	Astellas	Hinge-stabilized monospecific agonistic antibody	Fully human IgG4	No	Tetravalent
BMS-986156	Bristol-Myers Squibb	Monospecific agonistic antibody	Fully human IgG1	Yes	Not reported
GWN323	Novartis	Monospecific agonistic antibody	Humanized IgG1	Yes	Bivalent
INCAGN1876	Incyte	Monospecific agonistic antibody	Humanized IgG1	Yes	Not reported
MK-1248	Merck	Monospecific agonistic antibody	Humanized IgG4	No	Bivalent
MK-4166	Merck	Monospecific agonistic antibody	Humanized IgG1	Yes	Bivalent
REGN6569	Regeneron	Monospecific agonistic antibody	Fully human IgG1	Yes	Not reported
TRX518	Leap Therapeutics	Monospecific agonistic antibody	Fully humanized aglycosylated IgG1κ agonistic	No	Bivalent
Undisclosed	Abbvie	Anti-PD-1–GITR-L bispecific agonistic antibody	Humanized IgG1 with inert Fc	No	Trivalent
**Co-stimulatory GITR ligand**					
MEDI1873	AstraZeneca	Hexameric GITRL fusion protein (GITRL FP) comprising 2 GITRL ECD trimers and IgG1 Fc linked by isoleucine zipper trimerization domain	Fully human IgG1	Yes	Hexavalent
HERA-GITRL	Apogenix	Two trivalent single-chain GITRL binding domain (scGITRL-RBD) fused to IgG1 (Fc inert) dimerization scaffold	Humanized IgG1 with inert Fc	No	Hexavalent

**Table 2:** Reported clinical activity of GITR agonists being evaluated in ongoing or completed clinical trials

Agent (Sponsor)	Tumor type	Combination	Phase	Status	NCT.GOV ID	Single-agent activity	Combination activity	Primary publication
**Monospecific agonistic antibody**								
AMG-228 (Amgen)	All solid tumors	Not observed	I/II	Completed	NCT02437916	Not reported	Not applicable	Tran et al. [50]
ASP1951 (Astellas)	All solid tumors	Pembrolizumab	I/II	Active, not recruiting	NCT03799003	Not reported	Not reported	Not reported
BMS-986156 (Bristol-Myers Squibb)	All solid tumors	Nivolumab	I/II	Completed	NCT02598960	Not reported	Reported	Heinhuis et al. [47]
	Metastatic tumors in the liver or lung	Ipilimumab/nivolumab +/− SBRT	I/II	Active, not recruiting	NCT04021043	Not reported	Not reported	Not reported
GWN323 (Novartis)	All solid tumors and lymphomas	Spartalizumab	I/II	Completed	NCT02740270	Not reported	Reported	Piha-Paul et al. [49]
INCAGN1876 (Incyte)	All solid tumors	N/A	I/II	Completed	NCT02697591	Not reported	Not reported	Not reported
	All solid tumors	Ipilimumab or nivolumab	I/II	Completed	NCT03126110	Not reported	Not reported	Not reported
	Recurrent glioblastoma	Retifanlimab	II	Active, not recruiting	NCT04225039	Not reported	Not reported	Not reported
MK-1248 (Merck)	All solid tumors	Not observed	I/II	Completed	NCT02553499	Not reported	Reported	Geva et al. [46]
MK-4166 (Merck)	All solid tumors	Pembrolizumab	I	Completed	NCT02132754	Not reported	Reported	Papadopoulos et al. [48]
REGN6569 (Regeneron)	All solid tumors	Cemiplimab	I	Active, recruiting	NCT04465487	Not reported	Not reported	Not reported
TRX518 (Leap Therapeutics)	All solid tumors	Anti-PD-1 nivolumab, pembrolizumab and chemotherapy	I/II	Completed	NCT02628574	Reported	Reported	Davar et al. [45]
**Co-stimulatory GITR ligand**								
MEDI1873 (AstraZeneca)	All solid tumors	Not studied	I	Completed	NCT02583165	No objective responses (prolonged stable disease)	N/A	Balmanoukian et al. [44]

TNFRSF receptors such as GITR may also be activated by recombinant forms of their ligands. Fc-GITRL (MEDI1873) is one such example, and comprises 2 trimers of humanized GITRL extracellular domain (ECD) linked to two human IgG1 Fc domains by an isoleucine zipper trimerization domain that enforces ligand trimerization [54]. MEDI1873 demonstrated Fc/FcγR-mediated co-stimulatory activity and inhibition of Treg suppression in both *in vitro* and *in vivo* studies preclinically [54]. When evaluated in human cancer patients, MEDI1873 demonstrated an overall acceptable safety profile, reduced GITR+ Tregs within the tumor and dose-dependent GITR engagement on circulating memory T cells [45]. Despite its hexameric design and Fc receptor engagement, MEDI1873 produced only prolonged disease stabilization in patients and the lack of objective responses truncated its further clinical development [45].

To overcome the limitations of antibody based GITR agonists, Apogenix developed a novel hexavalent GITR agonist comprising two trivalent single-chain GITRL-receptor-binding-domain (scGITRL-RBD) units bound to an IgG1-derived silenced Fc-domain that serves as a dimerization scaffold (HERA-GITRL) [55]. Despite having no discernable effects upon Treg cell survival or proliferation, HERA-GITRL improved Teff function following stimulation *in vitro*. In syngeneic mouse tumor models, HERA-GITRL increased antigen-specific CD4+ and CD8+ T cell responses independent of FcγR-binding [55]. Clinical development plans of this agent have not been publicly reported.

Bispecific Abs targeting a co-stimulatory receptor and either an inhibitory immune receptor or a tumor-associated antigen (TAA) have the potential to localize the agonistic activity to immune cells or the tumor site, respectively. PD-1 and GITR are co-expressed on antigen-experienced T cells and memory T cells [44, 56]. In preclinical models, co-targeting PD-1 and GITR improves anti-tumor efficacy [43], possibly by restoring TIGIT/CD226 balance and preventing SHP2-mediated dephosphophorylation of the CD226 intracellular domain [44]. This has been explored with GITR in the context of an antibody-fusion protein composed of anti-PD-1 IgG1 antibody fused at the C-terminus of the silenced Fc to scGITRL [56]. The bispecific mAb induces FcγR-independent but PD-1 dependent GITR clustering in *cis*, resulting in enhanced activation, proliferation and memory differentiation of primed antigen-specific GITR+PD-1+ T cells along with consequent anti-tumor activity in syngeneic, genetically engineered and xenograft humanized mouse tumor models [56]. Compared to combination therapy with anti-PD-1 and anti-GITR (murine IgG2a with effector function), the bispecific mAb induces expansion of TAA-specific memory T cells with consequent rejection of tumor in challenge/rechallenge experiments [56]. However, it remains unclear whether this agent will be evaluated in human patients.

### Future directions

“*There is no perfect knowledge which can be entitled ours, that is innate; none but what has been obtained from experience, or derived in some way from our senses.*” – William Harvey. Lumleian Lecturer, and author, “De Motu Cordis”.

Overall, the clinical results obtained so far with GITR agonist agents have demonstrated specific immune effects in the expected immune cell populations based on preclinical studies. However, these effects have not produced substantial therapeutic activity in cancer patients. Our maturing understanding of the immune responses to GITR agonism in human cancers have clarified novel issues specific to drug development in this space including Ab structure (monospecific and bispecific mAbs and co-stimulatory GITR ligands), Ab valency, and Fc functionality. This improved understanding of the immune responses to GITR agonism in patients should be kept in consideration for the design of novel rational combinations or treatment regimens in earlier disease settings where immunotherapy is gradually becoming the treatment of choice. Considering the multiple mechanisms by which cancer cells can evade immune surveillance, having agents to modulate multiple immune targets concurrently may facilitate the design of therapeutic strategies that limit the development of resistance. In this direction, the experience with GITR targeting in patients may inform the development of next-generation immunotherapy approaches. Furthermore, these results underscore the mechanistic differences of critical immunotherapeutic pathways between preclinical animal models and humans. This suggests that advancing translational science for transformational impact in patients requires dedicated reverse translational efforts and the use of improved preclinical models.
